# Antiviral activity of ouabain against a Brazilian Zika virus strain

**DOI:** 10.1038/s41598-022-14243-5

**Published:** 2022-07-23

**Authors:** Deyse Cristina Madruga Carvalho, Poliana Gomes da Silva, Willyenne Marília Dantas, Severino Jefferson Ribeiro da Silva, Caroline Targino Alves da Silva, Elton José Ferreira Chaves, Demetrius Antônio Machado de Araújo, Ronaldo Nascimento de Oliveira, Sandra Rodrigues-Mascarenhas, Lindomar José Pena

**Affiliations:** 1 Laboratory of Immunobiotechnology, Biotechnology Center, Federal University of Paraiba (UFPB), João Pessoa, Paraiba Brazil; 2 Department of Virology and Experimental Therapy, Aggeu Magalhães Institute (IAM), Oswaldo Cruz Foundation (Fiocruz), Recife, Pernambuco Brazil; 3 Laboratory of Molecular and Cellular Biotechnology, Department of Biotechnology, Federal University of Paraiba (UFPB), João Pessoa, Paraiba Brazil; 4 Department of Chemistry, Federal Rural University of Pernambuco (UFRPE), Recife, Pernambuco Brazil

**Keywords:** Cell biology, Computational biology and bioinformatics, Drug discovery, Molecular biology

## Abstract

Zika virus (ZIKV) is an emerging arbovirus associated with neurological disorders. Currently, no specific vaccines or antivirals are available to treat the ZIKV infection. Ouabain, a cardiotonic steroid known as Na^+^/K^+^-ATPase inhibitor, has been previously described as an immunomodulatory substance by our group. Here, we evaluated for the first time the antiviral activity of this promising substance against a Brazilian ZIKV strain. Vero cells were treated with different concentrations of ouabain before and after the infection with ZIKV. The antiviral effect was evaluated by the TCID_50_ method and RT-qPCR. Ouabain presented a dose-dependent inhibitory effect against ZIKV, mainly when added post infection. The reduction of infectious virus was accompanied by a decrease in ZIKV RNA levels, suggesting that the mechanism of ZIKV inhibition by ouabain occurred at the replication step. In addition, our in silico data demonstrated a conformational stability and favorable binding free energy of ouabain in the biding sites of the NS5-RdRp and NS3-helicase proteins, which could be related to its mechanism of action. Taken together, these data demonstrate the antiviral activity of ouabain against a Brazilian ZIKV strain and evidence the potential of cardiotonic steroids as promising antiviral agents.

## Introduction

Zika virus (ZIKV) was first isolated in 1947 at the Zika forest in Uganda^1^. Since its identification, several cases of ZIKV infection in humans had been reported, but the virus only drew worldwide attention during the 2015–2016 epidemic in Brazil, when the association between ZIKV and microcephaly in neonates was first reported. This emerging virus is responsible to rash disease and also severe neurological manifestations in adults and neonates, such as Guillain–Barre syndrome in adults and congenital Zika syndrome (CZS)^2^. CZS is characterized by microcephaly, ocular anomalies, congenital contractures and other neurological lesions^3^. Since its emergence in the Americas, ZIKV has spread rapidly with the presence reported in 87 countries^2^.

ZIKV is an arthropod-borne virus (arbovirus) in the genus *Flavivirus* and the family *Flaviviridae,* which includes several other arboviruses of clinical importance (e.g., dengue virus [DENV], yellow fever virus [YFV] and West Nile virus [WNV]). Like the other flaviviruses, ZIKV is a positive-sense single-stranded RNA (+ _SS_RNA) virus with a genome size of approximately 11 kilobases. The RNA is translated into a single polyprotein encoding three structural proteins (capsid [C], precursor membrane [prM]/membrane [M] and envelope [E]) and seven nonstructural proteins (NS1, NS2A, NS2B, NS3, NS4A, NS4B and NS5). The structural proteins form the virus particle and mediate the initial steps of virus–host interaction, whereas the non-structural proteins assist in replication and packaging of the genome as well as evasion of immune defense mechanisms^4^. Several ZIKV proteins such as the envelope (E) protein, NS2-NS3 protease, NS3 helicase, NS5 methiltransferase and NS5 RNA-dependent RNA-polymerase (RdRp) have been implicated as potential targets for antiviral drugs. Molecular docking, a method that predicts interaction between proteins, has been widely used in the field of drug screening for in silico identification of antiviral candidates and also for postulating the mechanism of action of discovered antivirals^5^.

To date, no specific vaccines or drugs are available to treat ZIKV infection^2^. Moreover, the virus is still circulating in several regions of the world and could potentially cause new outbreaks; including in Brazil, where the African lineage was recently reported in the country in addition to the Asian lineage responsible for the 2015–2016 epidemics^6^. In this way, there is an urgent need for the development of safe and effective antiviral agents against ZIKV.

Cardiotonic steroids are natural compounds classically known to inhibit the Na^+^/K^+^-ATPase and to induce positive cardiac inotropism in high concentrations. However, these steroids not only inhibit the pump function but also activate intracellular signal transduction pathways, which are important in many biological processes^7^. Ouabain is a cardiotonic steroid originally described as plant-derived compound. In 1991, Hamlyn et al.^8^ reported the presence of ouabain in mammalian plasma. This steroid was considered a hormone and its physiological role has been studied since then^9^. Our group has described ouabain as an immunomodulatory substance, demonstrating its anti-inflammatory activity in vitro and in vivo models. This substance is capable to interfere with several inflammatory parameters in low concentrations, such as cell migration, vascular permeability and proinflammatory cytokines^10–14^.

In addition to its role in inflammation, the antiviral activity of ouabain has been reported in several studies^15–20^. Ouabain is effective against both DNA and RNA viruses at nanomolar concentrations^16,17^. This inhibitory effect can occur in different stages of the viral life cycle, such entry into the cell^18^ or interfering with the viral protein translation^19^. Therefore, the aim of this work was to evaluate the antiviral activity of ouabain against a Brazilian ZIKV strain in vitro and in silico.

## Results

### Cytotoxicity and antiviral activity of ouabain in Vero cells

**Figure 1:**
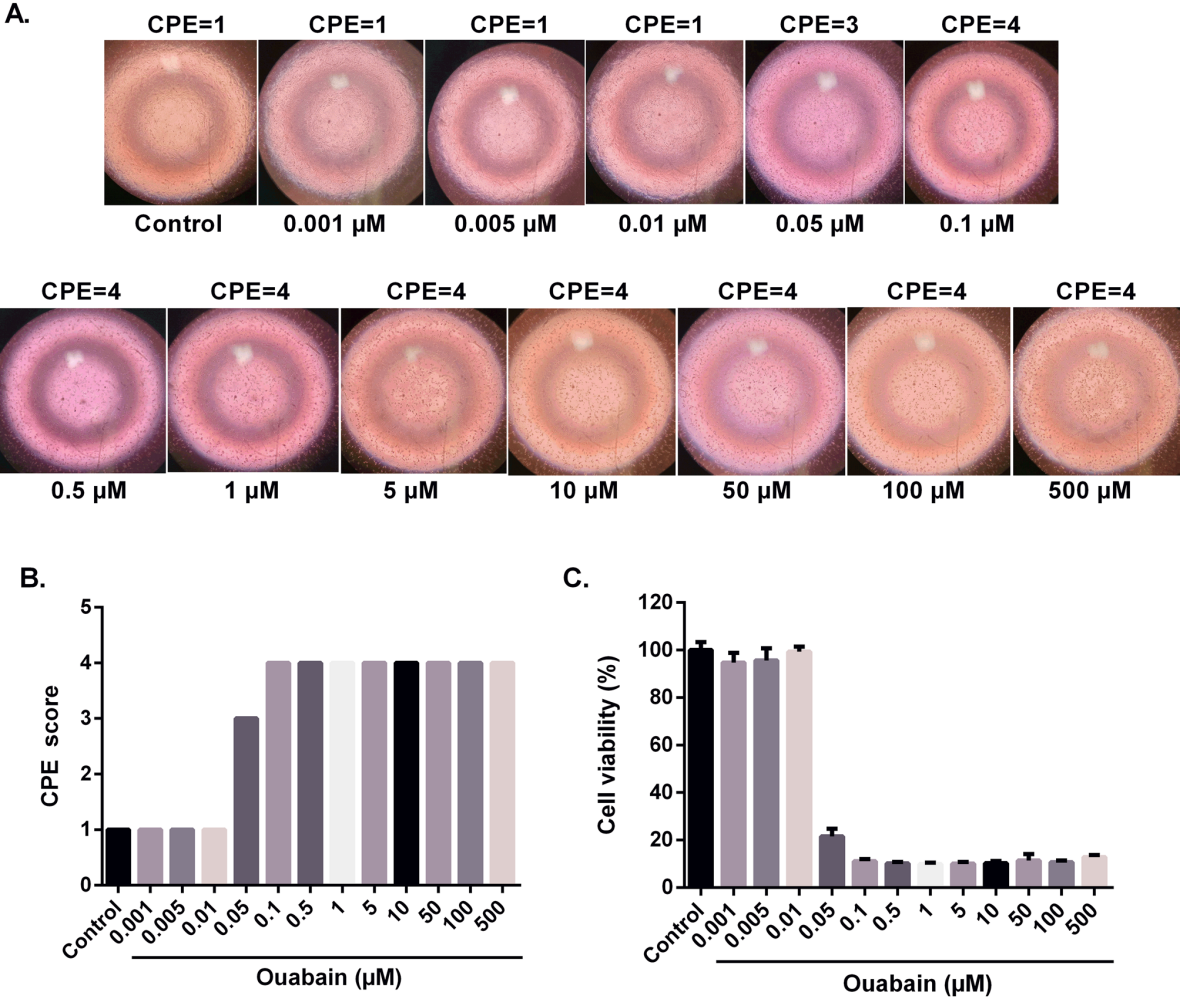
Cytotoxicity of ouabain. Vero cells were treated with different ouabain concentrations (n = 5). After 120 h of incubation at 37 °C, the CPE score was evaluated using an inverted microscope (**A**) and the CPE score was obtained (**B**). After that, the MTT solution (1 mg/mL) was added to each well and then the microplate was incubated for 4 h. The optical density was determined by spectrophotometry at 540 nm (**C**). MTT values were presented as mean ± standard deviation of three independent experiments. CPE values correspond to the CPE score means for replicates.

To assess the cytotoxicity and antiviral activity of ouabain against ZIKV, we determined its IC_50_, CC_50_, CC_20_ and SI for Vero cells upon treatment with various drug concentrations. As shown in Fig. 1 and Table 1, we found that its CC_20_ and CC_50_ values were at a nanomolar concentration, 20 and 68.9 nM, respectively. Thus, 20 nM (CC_20_) was defined as the maximal non-toxic concentration for antiviral screening^21^. The IC_50_ value of ouabain was also found at nanomolar concentration in both treatments (Pre-treatment—IC_50_ = 2.33 nM; Post-treatment—IC_50=_ 1.92). In addition, we calculated the SI of ouabain based on the effective drug concentration that results in the 50% virus inhibition (IC_50_) and the drug concentration that leads to 50% cytotoxicity (CC_50_). The SI (CC_50_/IC_50_) values found were 29.5 and 35.8, pre-treatment and post-treatment, respectively, demonstrating that the antiviral effects of ouabain are not related to cytotoxicity.

**Table 1:** Cytotoxicity (CC_50_ and CC_20_), antiviral potency (IC_50_) and selectivity index (SI) of ouabain.

Cell line	CC_50_ (nM)^a^	CC_20_ (nM)^b^	Pre-treatment		Post-treatment	
			IC_50_ (nM)^c^	SI^d^	IC_50_ (nM)^c^	SI^d^
Vero	68.9	20	2.33	29.5	1.92	35.8

^a^CC_50_ (50% cytotoxic concentration) refers to compound concentration that caused a 50% reduction in viability.

^b^CC_20_ (20% cytotoxic concentration) refers to the maximum non-toxic concentration employed in the antiviral assays.

^c^IC_50_ (50% inhibitory concentration) refers to compound concentration required to reduce viral titers by 50% compared with untreated controls.

^d^The Selectivity Index (SI) is obtained by calculating the ratio of the CC_50_ to the IC_50_.

### Ouabain treatment reduces ZIKV replication

**Figure 2:**
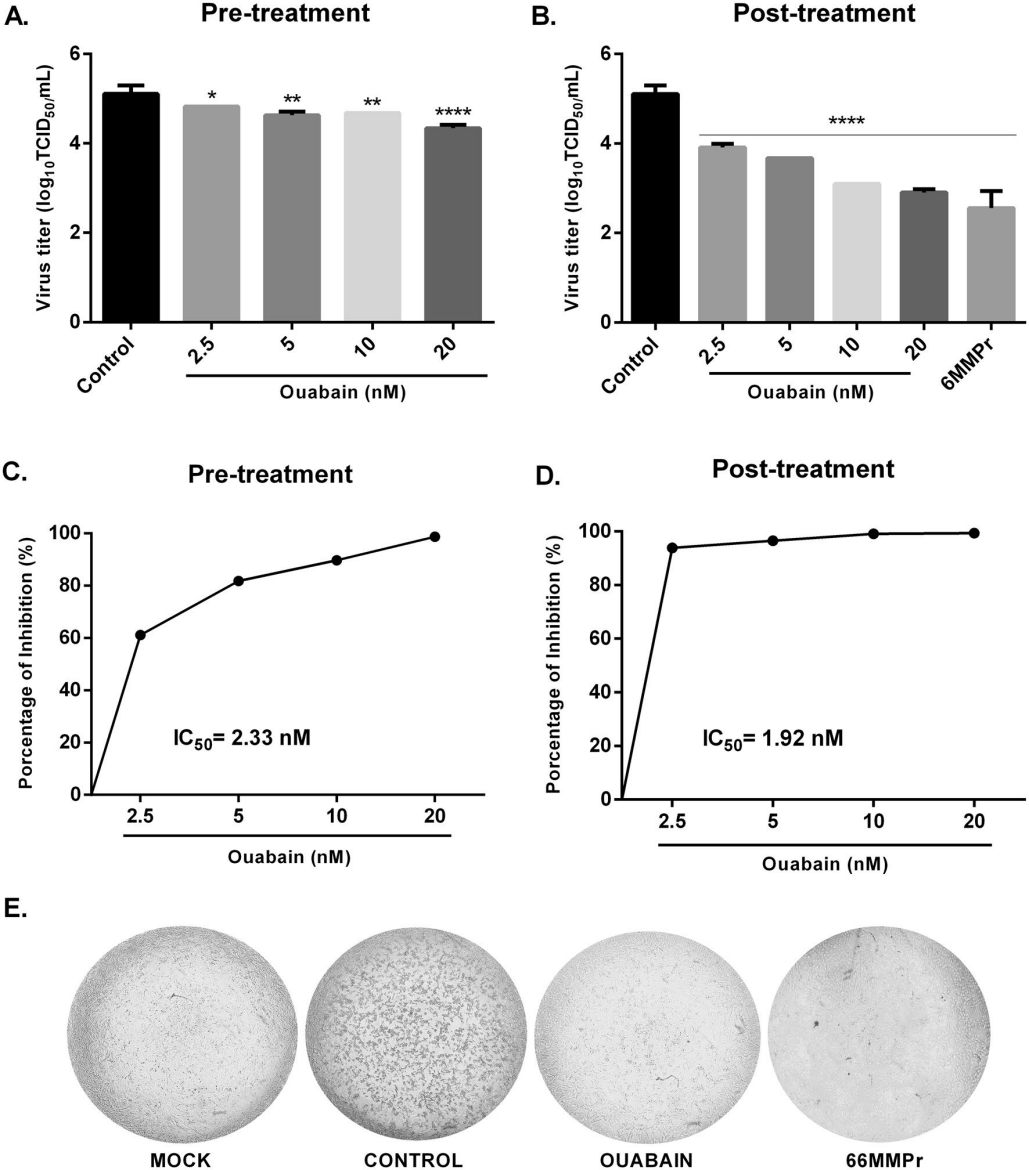
Ouabain reduced virus titer. Vero cells were treated with different concentrations of ouabain before (**A**,**C**,**E**) and after (**B**,**D**, 1 h of ZIKV infection with a multiplicity of infection (MOI) of 0.1. After 120 h, the cytophatic effect (**E**, was observed by optical microscopy and the supernatant was titrated using the TCID_50_ method. As a positive control, the antiviral compound 6MMPr (60.5 µM) was used in the post-treatment assay. Values were presented as mean ± standard deviation of three independent experiments and analyzed by one-way analysis of variance (ANOVA) followed by Tukey post-test. *P < 0.05, **P < 0.01, ***P< 0.001, ****P < 0.0001 significant in relation to the control.

As shown in Fig. 2, ouabain was capable of reducing live virus titer when compared to the control in both assays (Fig. 2A,B). This reduction was observed in all concentrations tested with a percentage decrease of 61.2%, 81.8%, 89.7% and 98% in the pre-treatment assay and 93.7%, 96.3%, 99% and 99.3% in the post-treatment at concentrations of 2.5, 5, 10 and 20 nM, respectively (Fig. 2C,D). The highest concentration in post infection assay (Fig. 2B) decreased virus titer with a reduction to 2.2 log_10_ units in comparison to the control, which is similar to the reduction achieved with positive control^21^, 6MMPr (2.5 log_10_, 99,7% reduction). In addition, this concentration also prevented morphological changes associated with the progression of infection (shrinkage and clumping) that characterizes the ZIKV cytopathic effect, as can be seen in Fig. 2E.

### Ouabain treatment reduces ZIKV RNA copy numbers

**Figure 3:**
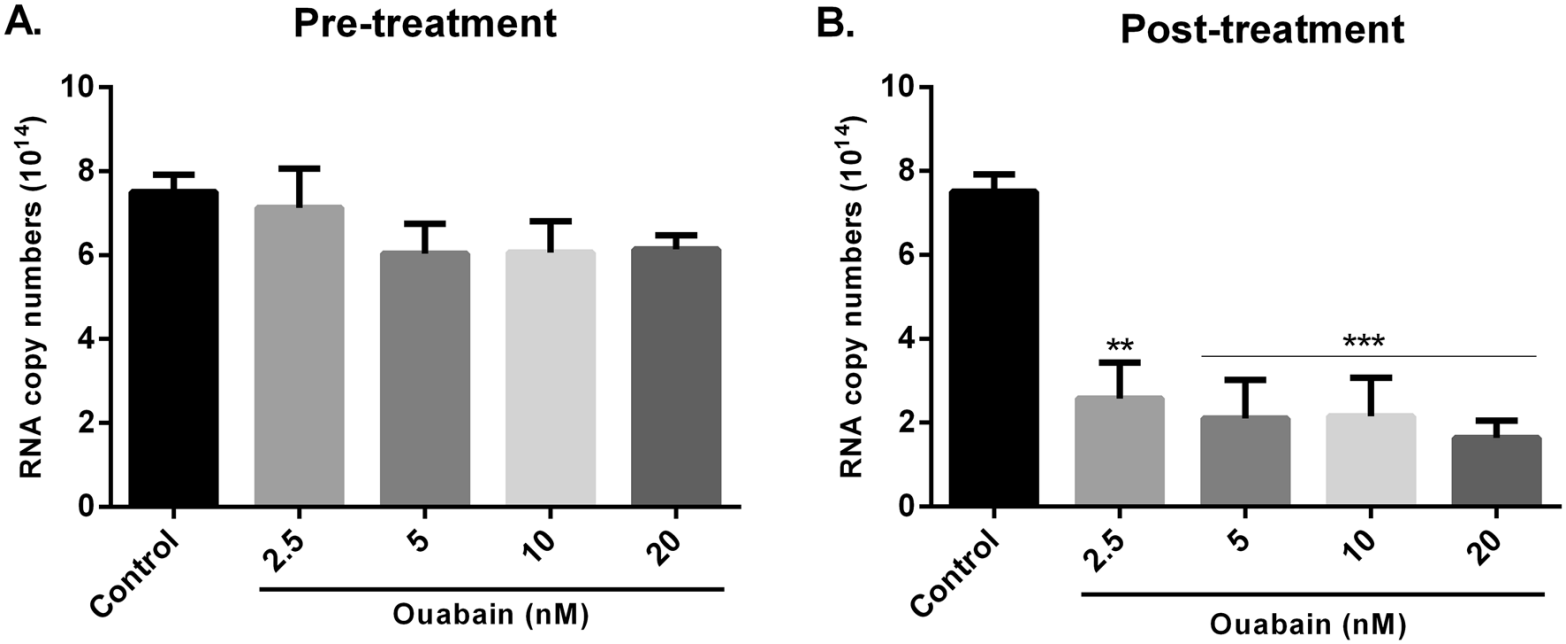
Ouabain decreased the RNA copy numbers only in the post-treatment assay. Vero cells were treated with different concentrations of ouabain before (**A**) and after (**B**) 1 h of ZIKV infection with a multiplicity of infection (MOI) of 0.1. After 120 h, the supernatant was collected and the RNA copy numbers were quantified by reverse transcription quantitative PCR (RT-qPCR). The RNA copy numbers were calculated using a standard curve. Values were presented as mean ± standard deviation of three independent experiments and analyzed by one-way analysis of variance (ANOVA) followed by Tukey post-test. **P < 0.01, ***P< 0.001 significant in relation to the control.

As shown in Fig. 3A, ouabain pre-treatment did not interfere with the RNA copy numbers in comparison to the control group. On the other hand, post-treatment of ouabain was effective in reducing these RNA levels (Fig. 3B). This reduction was observed in all concentrations tested with a percentage decrease by 65.6%, 71.9%, 71.3% and 78.1% at concentrations of 2.5, 5, 10 and 20 nM, respectively.

### Ouabain did not impair the initial step of the virus life cycle

**Figure 4:**
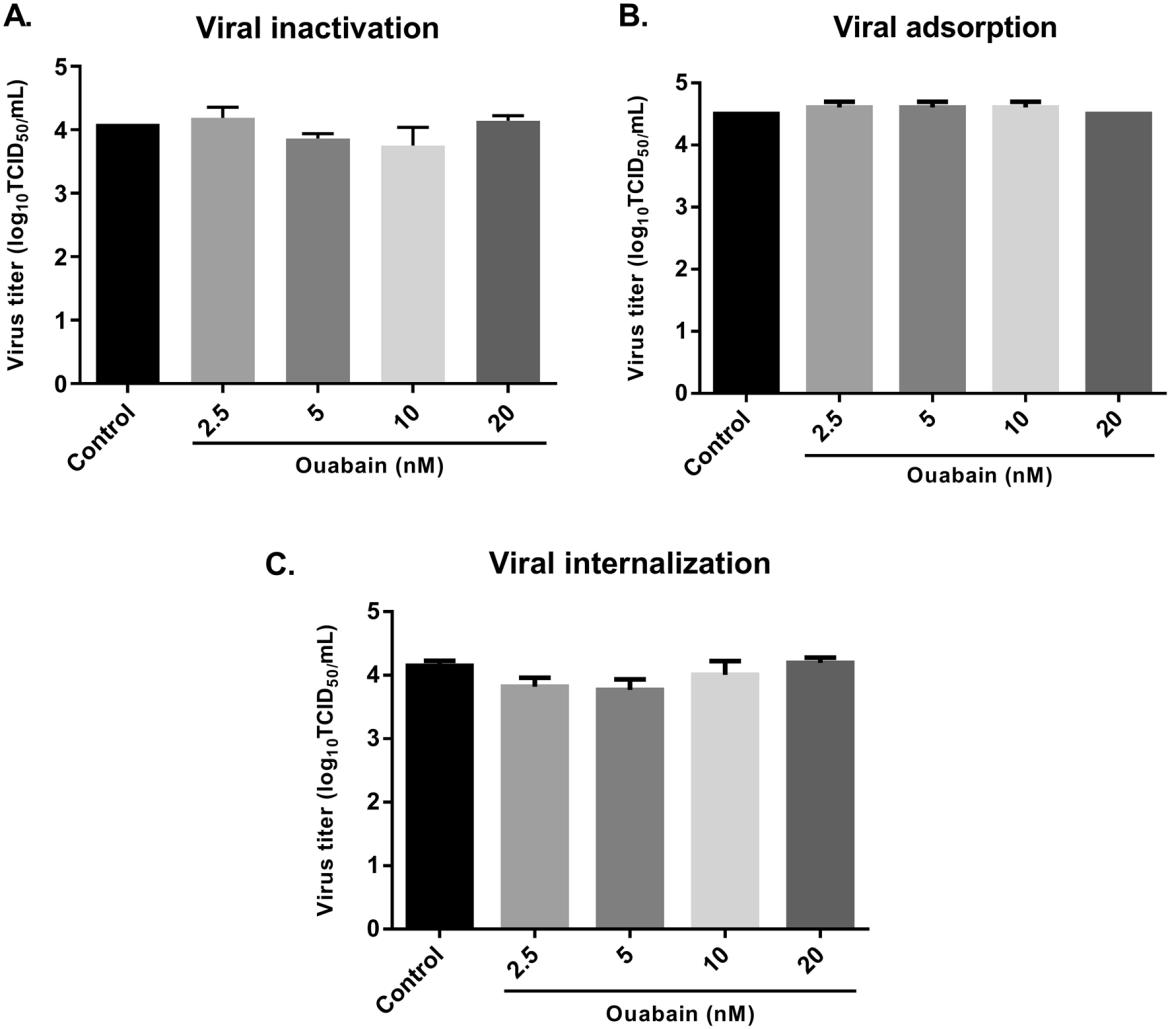
Ouabain did not impair the entry of the virus into the cell. (**A**) A viral suspension containing ZIKV at multiplicity of infection (MOI) of 0.1 was incubated with equal volume of different concentrations of ouabain for 1 h at 37 °C to evaluate the virucidal activity of ouabain. Then, the suspension was titrated using the TCID_50_ method. (**B**) Vero cells were infected with ZIKV (MOI = 0.1) and treated with different concentrations of ouabain for 1 h at 4C for virus adsorption. After 120 h, the supernatant was collected and titrated using the TCID_50_ method. (**C**) Vero cells were incubated at 4 °C for 1 h to virus attachment (MOI = 0.1). After 1 h of adsorption, unabsorbed viruses were removed and cells were incubated at 37 °C for 1 h with different concentrations of ouabain. Then, cells were treated with 0.1 ml of citrate buffer for 1 min to inactivate adsorbed viruses but not internalized. After 120 h, the supernatant was collected and titrated using the TCID_50_ method. Values were presented as mean ± standard deviation of three independent experiments and analyzed by one-way analysis of variance (ANOVA) followed by Tukey post-test.

To determine whether ouabain interferes with the entry of the virus into the cell, a time-of-addition experiment was performed. As shown in Fig. 4A, ouabain did not present direct virucidal activity on ZIKV particles. Incubation of virus with ouabain in different concentrations (2.5, 5, 10 and 20 nM) prior to the cells had no effect on viral titers (Fig. 4A), demonstrating that the antiviral activity of this cardiotonic steroid is not attributable to inactivation of the virus.

Next, we investigated the effect of ouabain against virus attachment to the host cell. Our data showed that ouabain did not interfere with adsorption or internalization of the virus. Different ouabain concentrations (2.5, 5, 10 and 20 nM) had no effect on viral titer when compared to the control group in both assays (Fig. 4B,C).

### Binding pose analysis and relative binding affinity between ouabain and the main drug targets of ZIKV

**Figure 5:**
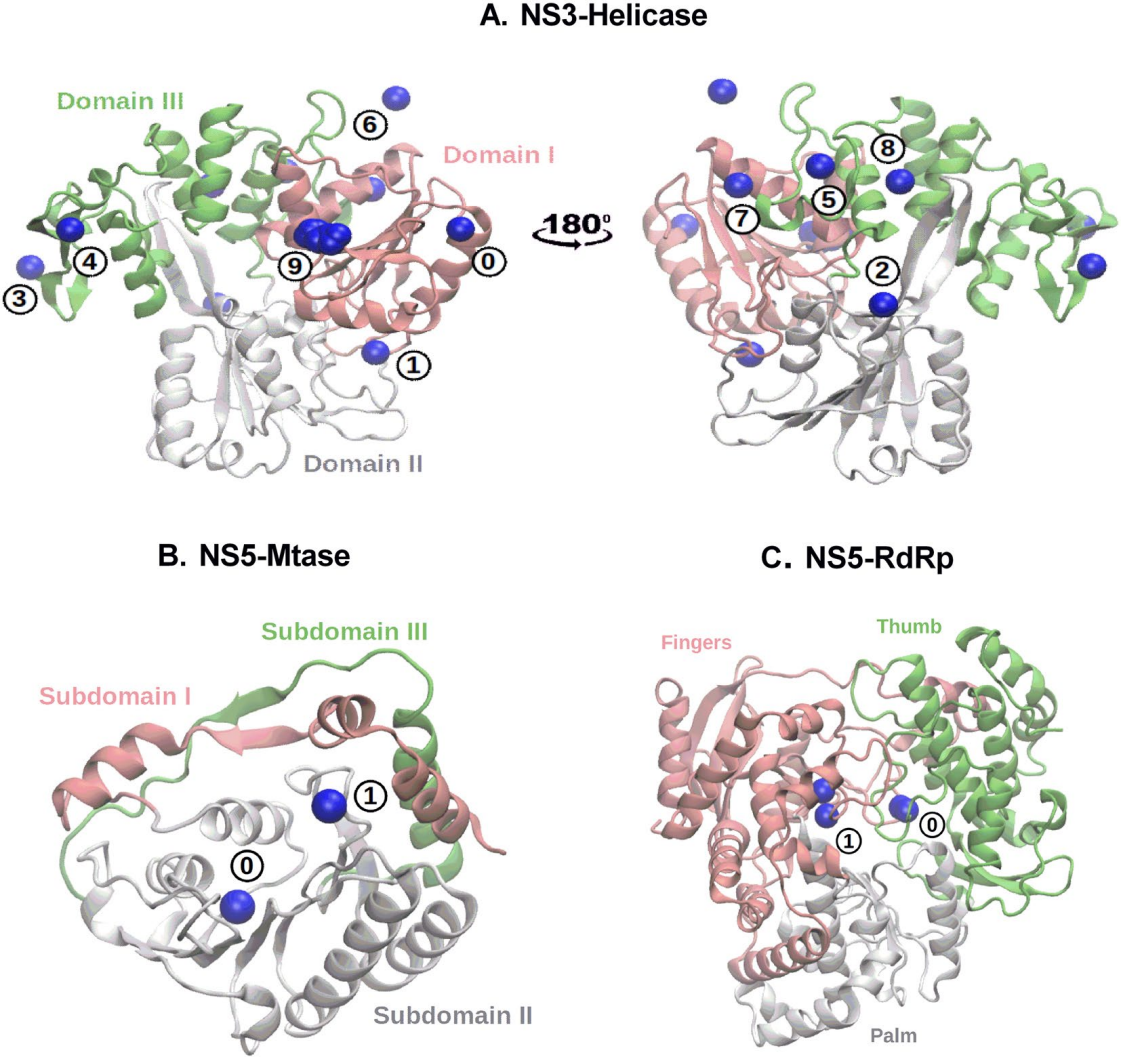
Binding pockets of the three non-structural ZIKV proteins used in this study: (**A**) NS3-Helicase, (**B**) NS5-Mtase and (**C**) NS5-RdRp. The blue sphere indicates the center of mass of the small-molecules that have been co-crystallized with these targets. Domains and subdomains of each protein are depicted in green, pink and white colors.

In order to assess the ability of ouabain to interact with non-structural ZIKV proteins, we performed a molecular docking procedure followed by molecular dynamics simulations. The following NSPs of ZIKV were used: NS3-Helicase, NS5-Mtase, and NS5-RdRp. As first step, we looked for druggable binding cavities using the chemical information of small-molecules that were co-crystallized with these receptors, and we found a total of 10, 2 and 2 binding sites in NS3-Helicase, NS5-Mtase, and NS5-RdRp, respectively. A representation of these cavities is depicted in Fig. 5.

Upon identification of the druggable binding cavities, it was possible to test whether ouabain can interact with the same region of these proteins. Therefore, we used this biological information to guide our molecular docking protocol to predict the ouabain binding pose. Table 2 presents the docking scores (*E*_*docking*_) for ouabain at the different binding sites. Molecular docking pointed out that ouabain interacts with high affinity at the binding site 1 of NS5-Mtase, followed by the NS5-Mtase binding site 0 and NS3-Helicase binding site 7. The GA of the GOLD software was not able to produce binding poses of the ouabain in the NS5-RdRp binding site 0, perhaps, because a steric hindrance in this moiety of the protein. Further, the ouabain binding pose at the binding site 1 of this receptor showed a very low docking score (Table 2).

**Table 2:** Prediction of the relative binding affinity between ouabain and the binding pockets of non-structural ZIKV proteins. The third, fourth, and fifth columns comprise the score obtained in the molecular docking step, free energy of binding (in kcal/mol) obtained by the MM/PPBSA method, and binding enthalpy (in kcal/mol), respectively.

Molecular target	Binding site (BS)	*E*_*docking*_	∆*G*_*bind*_	∆*H*_*bind*_
NS3-Helicase	0	17.94	− 1.92 ± 0.65	− 90.55 ± 10.35
	1	24.03	− 4.16 ± 0.58	− 109.93 ± 11.09
	2	17.21	− 0.71 ± 0.46	− 83.80 ± 13.94
	3	17.01	− 4.44 ± 0.60	− 94.55 ± 12.07
	4	22.78	− 2.18 ± 0.37	− 89.79 ± 13.31
	5	20.77	− 9.48 ± 0.44	− 117.30 ± 10.32
	6	18.89	− 1.90 ± 0.56	− 86.59 ± 9.08
	7	24.04	− 5.54 ± 0.55	− 106.66 ± 8.16
	8	19.76	− 0.92 ± 0.57	− 85.86 ± 14.28
	9	19.23	− 5.11 ± 0.35	− 94.61 ± 14.57
NS5-Mtase	0	25.63	− 4.79 ± 0.52	− 116.68 ± 13.97
	1	27.09	− 0.95 ± 0.35	− 76.64 ± 12.62
NS5-RdRp	0	–	–	–
	1	17.38	− 10.58 ± 0.77	− 121.76 ± 14.21

### Post-docking analysis showed that ouabain interacts with the RdRp domain of the NS5 protein and the binding site 5 of the NS3-Helicase

**Figure 6:**
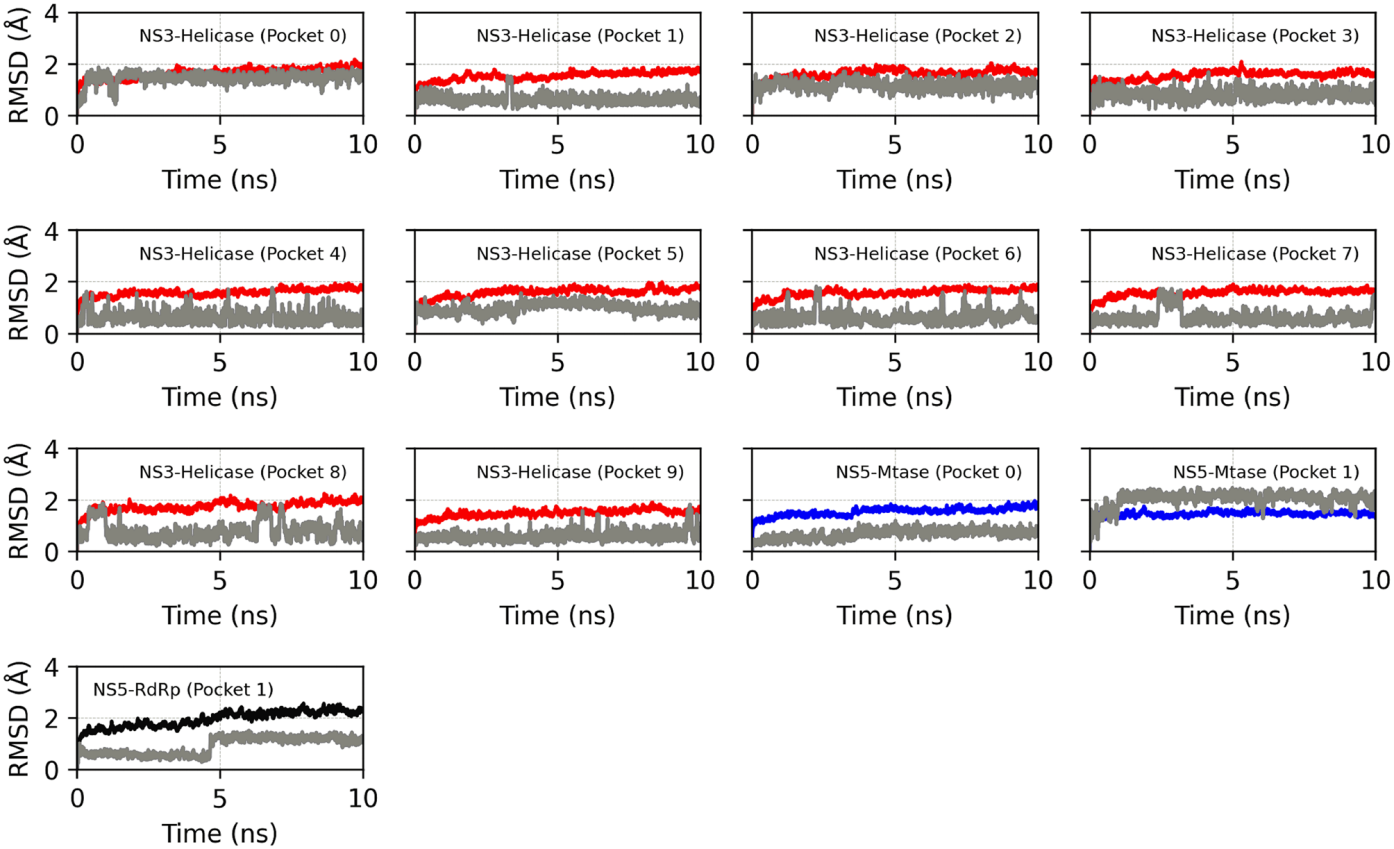
RMSD profile of the NSP-ouabain complexes. In red, blue and black colors are depicted the RMSD profiles of the NS3-Helicase, NS5-Mtase, and NS5-RdRp segments, when attached to ouabain in different binding pockets; the RMSD profiles of the ouabain segment is depicted using gray color. For the segment relative to protein, RMSD was calculated considering backbone atoms, whereas ligand was computed considering only heavy atoms.

The NSP-ligand complexes obtained in the molecular docking step were subjected to molecular dynamics simulations to assess the stability of ouabain, as well as to re-scoring the relative binding affinity using the MM/PBSA method and semi-empirical quantum calculations. Figure 6 shown the RMSD profiles of the NSP-ouabain complexes obtained from the trajectory of MD simulation. We observed that the backbone of NS3-Helicase, NS5-Mtase, and NS5-RdRp did not undergo conformation changes along the time when in complex with ouabain at different binding sites. Further, considering the last 5 ns of MD simulations, it was possible to see that the ouabain achieved a conformational stability in the binding sites 0, 1, 5, and 7 of NS3-helicase. Regarding the NS5-Mtase/ouabain and NS5-RdRp/ouabain complexes, the starting geometry of the ouabain at the binding pocket 1 of the Mtase and RdRp domains (NS5) underwent conformational changes at the begining of the MD simulation. However, after 5 ns of MD simulation, the ouabain segment reacheds a stable conformation.

**Figure 7:**
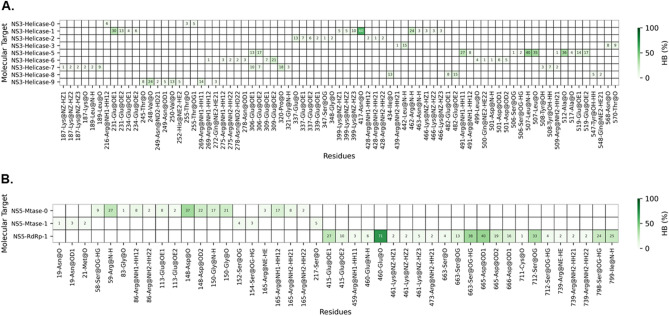
Heatmap showing the hydrogen bonds profile between ouabain when bound at different binding sites of non-structural ZIKV proteins. (**A**) NS3-Helicase; (**B**) NS5. The x-axis should read as follows: residue position (number)—Residue name @ hydrogen bond donor or acceptor.

**Figure 8:**
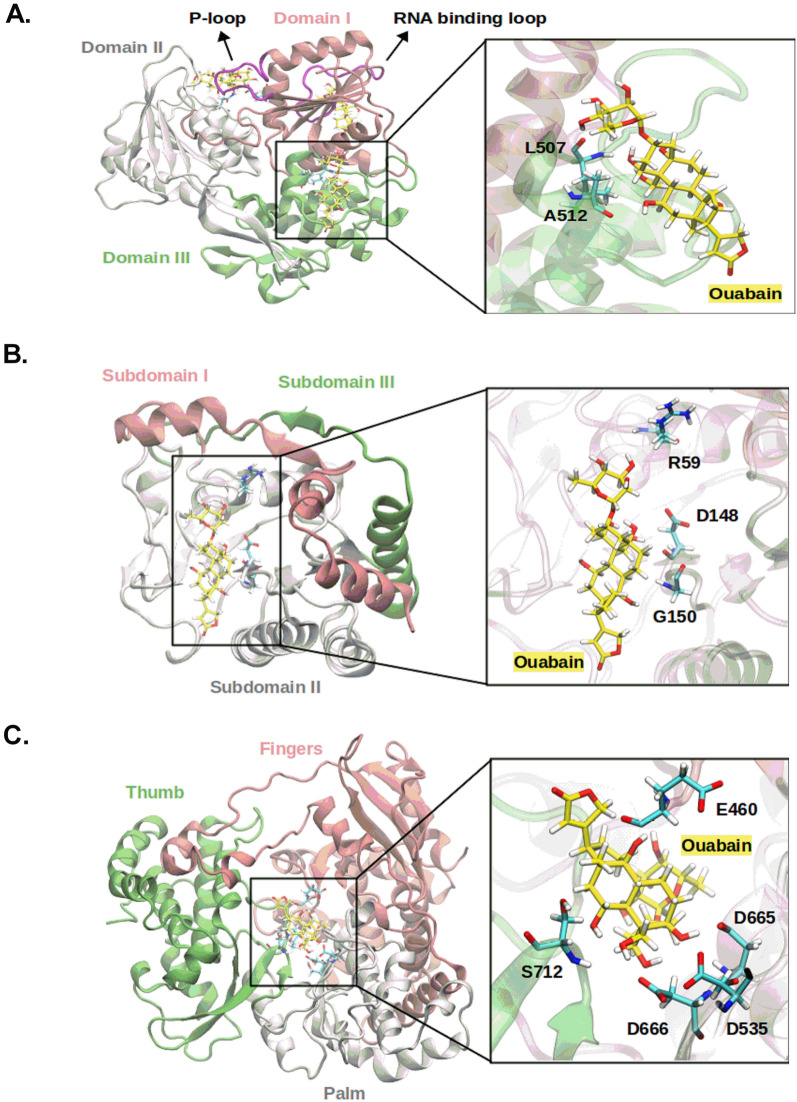
Binding poses of ouabain at the binding sites of non-structural ZIKV proteins. Receptor residues are depicted in cyan color, while ouabain carbon atoms are depicted in yellow color. (**A**) NS3-Helicase, (**B**) NS5-Mtase and (**C**) NS5-RdRp.

The relative binding affinity predicted by MM/PBSA method showed that the ouabain has a high affinity for the binding site 1 of NS5-RdRp, ∆*G*_*bind*_ = − 10.58 kcal mol^−1^, followed by NS3-Helicase binding site 5, ∆*G*_*bind*_ = − 9.48 kcal mol^−1^ (Table 2). Similarly, the calculation of the enthalpy of binding (∆H_*bind*_) showed that ouabain has a high affinity for the binding site 1 of NS5-RdRp, ∆H_*bind*_ = − 121.76 kcal mol^−1^, followed by NS3-Helicase, ∆H_*bind*_ = − 117.30 kcal mol^−1^ (Table 2). The Pearson correlation coefficient (R^2^) between the relative affinities calculated by the MM/PBSA method and semi-empirical quantum calculations was 0.77. Figure 7 showed the occurrence of hydrogen bonds between ouabain and the binding sites that achieved ∆*G*_*bind*_ ≤ − 4.0 kcal mol^−1^ (Table 2). At the binding site 5 of NS3-helicase protein, a hydrogen bond between the oxygen and nitrogen backbone atoms of Leu507 and the ouabain seemed to play an important role for the ouabain stability (Figs. 7A, 8A). Also, the oxygen backbone atom of Ala517 formed a hydrogen bond with ouabain. Furthermore, considering an occurrence percentage greater than 30% as a "hot spot" for hydrogen bond with ouabain, the following residues of NS3-Helicase can be highlighted: Glu231 and Asn417 (Fig. 7A). Regarding the NS5 protein, the NS5-Mtase domain did not form strong hydrogen bonds with ouabain along the MD simulation (Figs. 7B, 8B). In contrast, the residues Glu460, Ser663, Asp6651, and Ser712 from the NS5-RdRp domain form strong hydrogen bonds with ouabain, being Glu186 the most important one (Figs. 7B, 8C).

## Discussion

The explosive epidemics of ZIKV in Brazil and other Latin America countries in 2015–2016 and the recent Indian outbreak highlight the potential of ZIKV for rapid spread in the population. Despite the severe consequences of ZIKV infection, no specific vaccines or antivirals are currently available to treat the associated diseases^2^. Cardiotonic steroids, which are used in patients with congestive heart failure, have been investigated to treat other diseases, such as cancer^22^ and viral infection^15^. In this work, we demonstrate the potential of the cardiotonic steroid ouabain against a Brazilian ZIKV strain isolated during the 2015 epidemic in Brazil^23^.

Ouabain has antiviral activity against several RNA and DNA viruses, such as cytomegalovirus^17^, herpes simplex virus^24^, influenza virus^19^, human immunodeficiency virus^16^, Japanese encephalitis virus^25^ and coronaviruses^18^, including the severe acute respiratory syndrome coronavirus 2 (SARS-CoV-2)^26^. By the time of writing this manuscript, the effect of ouabain against two ZIKV strains (ZIKV H/PF/2013 from French Polynesia and ZIKV MRS from Martinique) was described by Guo et al.^20^. Therefore, here we report our independent findings of ouabain inhibition of a Brazilian ZIKV strain and discuss similarities and differences between these studies.

Our data showed that ouabain was capable to reduce viral titer at nanomolar concentrations, concurring with several previous studies^16–19^. This inhibitory effect was observed in both pre-treatment and post-treatment assays. However, the treatment of ouabain after the infection was more effective to reduce ZIKV titer with a decreased up to 2.2 log_10_ units (99.3%) at the highest concentration, similar to the positive control 6MMPr, a drug previously identified by our group as a potent ZIKV inhibitor^21^. Additionally, we evaluated the RNA copy numbers and observed that ouabain was capable to reduce viral RNA levels. This effect on viral RNA was seen only in the post-treatment assay, corroborating the results obtained with virus titration. Notably, these data indicate that this substance shows effective antiviral activity against ZIKV mostly when administered post infection, in agreement with the data published by Guo et al.^20^. Although the IC_50_ value in both studies was at a nanomolar range, we reported the ouabain effect using a concentration approximately 50-fold lower than showed by them^20^, demonstrating the potential of ouabain against ZIKV at lower concentrations. The Brazilian ZIKV strain (ZIKV/Brazil/PE243/2015) is representative of the ZIKV that caused the epidemics in Brazil (2015–2016), which was associated with the development of severe birth defects, including microcephaly in neonates, and neurological diseases such as Guillain-Barré Syndrome (GBS) in adults^23,27^. This strain demonstrates a strong degree of conservation at amino acid level (98.3% pairwise identity) with sequences from 62 isolates, included the strains used by Guo et al.^20^. However, although the level of genetic similarity, strains could present differences in the percentages of infected cells, RNA accumulation and/or viral progeny, as previously described^28,29^. In this way, the differences between the ZIKV strains used in both studies may have accounted for the observed variation on ouabain concentration.

Ouabain has been shown to interfere both in the entry (e.g., against coronaviruses)^18^ and in the replication stage (e.g., against influenza virus)^19^ of the virus life cycle. To get more insight into the antiviral effect against ZIKV, we analyzed whether the action of ouabain could be related to the entry stage. We found that this substance is not capable to block the virus adsorption or internalization steps, nor it can directly inactivate the virus (virucidal activity). Together, these data suggest that ouabain may not interfere in the entry, but seems to act in the post entry stage of the ZIKV life cycle. This hypothesis is supported by previous results where the substance showed strong inhibitory effects when added post infection. In addition, the replication step appears to be the main mechanism by which cardiotonic steroids demonstrate their antiviral activity, as reported by Amarelle and Lecuona^15^. Here, we found that ouabain was capable of reducing ZIKV RNA copy numbers, suggesting an action of ouabain at the replication stage and this was corroborated by Guo et al.^20^.

The antiviral effect of cardiotonic steroids has been attributed to binding to their receptor, Na^+^/K^+^-ATPase^18–20^. This binding can result in changes in the intracellular concentrations of sodium, potassium, and calcium and/or also trigger signaling transduction pathways^7^. It has been reported that these both mechanisms are involved in the activity of steroids cardiotonic on viral replication^15^. However, Mastrangelo and colleagues demonstrated that ouabain could also have a direct action on viral protein^30^. To answer this hypothesis, we performed a molecular docking procedure followed by molecular dynamics simulations to predict the interaction between ouabain and the main drug targets in ZIKV proteome (NS3 Helicase, NS5 Mtase and NS5 RdRp). Our data indicated that ouabain possibly interacts in the binding site 5 of NS3-Helicase, ∆*G*_*bind*_^MM/PBSA^ = − 9*.*48 kcal mol^−1^. Furthermore, ouabain also showed favorable binding energies (ΔG_bind_^MM/PBSA^ < − 4 kcal/mol) in the binding sites close to important segments of the enzyme, such as the loop regions related to ATP hydrolysis (P-loop) and RNA binding (RNA binding domain) (Fig. 8A). Kumar et al.^31^ evaluated the antiviral potential of the polyphenol EGCG (PubChem ID: 65064) in the NS3-helicase of ZIKV. Molecular docking and DM simulation showed that EGCG form hydrogen bonds at the ATPase site, mainly with N417, as well as in the RNA binding site. In vitro assays showed an inhibition of NTPase activity with IC_50_ of 295.7 nM. The hydrogen bond analysis performed in our study showed that ouabain interacts strongly with the same residue, N417, near to the P-loop. Therefore, it is possible that ouabain is inhibiting ZIKV viral replication through an allosteric effect of the NS3-helicase enzyme. Additionally, our data corroborate the findings made by Mastrangelo et al.^30^, who found that ouabain presented a ∆G for NS3 helicase domains of West Nile Virus (WNV) ranging from − 11.5 to − 9.5 kcal mol^−1^. In addition, the authors demonstrated that ouabain inhibited the dsNRA unwinding activity of WNV helicase in vitro, evidencing ouabain as a potential flavivirus helicase binding compound^30^.

Moreover, our in silico data also indicated that ouabain possibly interacts with the RdRp domain of the NS5 protein, ∆*G*_*bind*_^MM/PBSA^ = − 10*.*58 kcal mol^−1^ (Table 2);* ∆H*_*bind*_ = − 121.76 kcal mol^−1^ (Table 2). Pattnaik et al.^32^ by using molecular docking approach showed that the TPB compound (PubChem ID: 1619825) attach in the NS5-RdRp active site and forms hydrogen bonds with residues D535 and D665. Cell-based assays showed that TPB had IC_50_ = 94 nM. Our in silico results showed that ouabain also interacts close to the active site of RdRp (site 1), with residues E460, D665, and D666. These residues (D535, D665, and D666) are highly conserved in the active site of RdRps of flaviviruses and play an important role in the polymerization of the RNA strand. In this way, our results suggest that ouabain may be preventing the replication of ZIKV through the allosteric effect of the RdRp enzyme. Therefore, the stability and longer residence time in the binding sites of NS3-helicase and NS5 RdRp could be related to the inhibition of the activity of these virus proteins by ouabain. The NS3 helicase provides chemical energy to unwind viral RNA replication intermediates. This facilitates the replication of the viral genome, promoted in concert with the NS5 RdRp^31,33^. Therefore, these proteins play essential role in the viral life cycle and their inhibition results in deficiency of production of viral particles^5^. Depth biochemical studies characterizing the interaction of ouabain with individual ZIKV proteins are warranted and will shed light on its mechanisms of action against viruses.

The in silico data reveal new insights about the targets of ouabain during the ZIKV infection. Although the classical explanation for the multiple effects of cardiac steroids has been through the Na^+^/K^+^-ATPase, other targets cannot be excluded. The works of Valente et al.^34^ and Alonso et al.^35^ demonstrated the ability of fluorescent analog OUABDP easily reaches the cytoplasm of Ma104 cells and HeLa cells, respectively. The Valente’s work raises the possibility that this substance may conceivably reach the cytoplasm and probably binding to steroid receptors or, alternatively, to its own intracellular (yet unknown) receptor. In addition, the Alonso’s work observed that ouabain binds to Na^+^/K^+^-ATPase and the complex formed is internalized and co-localizes later on with the mitochondria, indicating the existence of a mitochondrial binding site for the ouabain-Na^+^/K^+^-ATPase complex. Together, these works suggested that ouabain could have binding sites inside the cell, however further studies are need to better understand this real possibility.

Drug repositioning, a strategy for identifying new uses for approved drugs, is increasingly becoming an attractive proposition due to the *reduce* drug *development cost* and time^36^. Cardiotonic steroids, used in patients with congestive heart failure, has been suggested to present other therapeutic effects. Ouabain has been described as a potential candidate to treat viral infections, due its capacity of targeting cell host proteins, which help to minimize resistance and also its effectiveness against a broad spectrum of virus species^15^. Here, we demonstrated for the first time the antiviral activity of this cardiotonic steroid against a Brazilian ZIKV strain, making this compound an attractive candidate for further in vivo studies aimed at finding effective antivirals against ZIKV.

In addition to the action as drugs, cardiotonic steroids were identified in human fluids and tissues by the end of the past century^8^. The endogenous ouabain has been widely studied for its role in physiology. It has known that ouabain levels in the plasma vary from the picomolar to the nanomolar range, nevertheless there are physiological situations, such as pregnancy and intensive exercise, that increased these levels^37^. Moreover, endogenous ouabain has been described as an immunomodulatory substance^10–14^ and as a modulator of neuroinflammation^38^. Considering that ZIKV infection causes intense inflammatory response in the brain and ouabain present anti-inflammatory and antiviral activity, a question arises: could endogenous ouabain be able to inhibit or modulates ZIKV infection in humans? Further studies are needed to understand whether this hormone could be acting as physiological antiviral agent.

## Conclusions

In conclusion, our findings demonstrated the antiviral activity of ouabain against a Brazilian ZIKV strain for the first time, concurring the previous data with other ZIKV lineages. Moreover, our data in silico revealed new insights about the targets of ouabain during the ZIKV infection. Finally, this study contributes to confirm the effectiveness of cardiotonic steroids as promising antiviral agents.

## Materials and methods

### In vitro experiments

#### Cells and virus

Vero cells were grown in Dulbecco’s modified Eagle’s medium (DMEM) (Gibco, Carlsbad, CA) supplemented with 10% inactivated fetal bovine serum (FBS) (Gibco), 2 mM l-glutamine (Gibco) and 100 U/mL penicillin/streptomycin (Gibco). The Brazilian ZIKV strain, named ZIKV/*H.sapiens*/Brazil/PE243/2015 (abbreviated to ZIKV PE243; GenBank accession no.KX197192.1), was isolated from a patient with the classical ZIKV exanthematous illness without neurological signs and used throughout the study^23^. The PE243 strain was propagated and titrated on Vero cells by fifty-percent tissue culture infection dose (TCID_50_) method^39^ using cytopathic effect (CPE) as the readout.

#### Cell viability assay

The cytotoxicity of ouabain (Sigma-Aldrich, O3125) was tested by MTT (3-[4,5-dimethylthiazol-2-yl]-2,5-diphenyltetrazolium bromide) assay (Sigma) and by CPE (cytopathic effect) score. Briefly, Vero cells were seeded into 96-well plates at a density (1 × 10^4^ cells/well) that allowed 80–90% confluence to be reached within 24 h at 37 °C with 5% CO_2_. Then, cells were treated with increasing concentrations of ouabain (0.001, 0.005, 0.01, 0.05, 0.1, 0.5, 1, 5, 10, 50, 100, 500 μM). After 120 h of incubation at 37 °C in a 5% CO_2_ atmosphere, the CPE score was evaluated using an inverted microscope (AE2000 binocular microscope, Motic, Hong Kong). The CPE scores were defined as (1) CPE < 25%, (2) CPE between 25 and 50%, (3) CPE between 51 and 75% and (4) CPE > 75%, as described by Carvalho et al.^40^. After that, 50 μL of freshly prepared MTT solution (1 mg/mL) was added to each well and then the microplate was incubated for 4 h_._ Then, MTT formazan crystals were solubilized by adding dimethyl sulfoxide (DMSO) and the optical density at 540 nm (OD_540_) was determined spectrophotometrically using the BioTek™ ELx800™ 96-well plate reader (BioTek Instruments Inc., Winooski, VT). Cell viability was calculated by subtracting the OD_540_ of treated cells from untreated cells.

#### Viral titration

Vero cells were cultivated in 96 well plates at the density of 1 × 10^4^ cells/well at 37 °C in a 5% CO_2_ incubator one day prior to titration. Supernatants from antiviral assays were tenfold serially diluted in DMEM. The diluted supernatant was then added to the cells, which were further incubated for 5 days at 37 °C and 5% CO_2_. The cytopathic effect was evaluated on an inverted optical microscope and the reduction of viral titer was expressed as log_10_ TCID_50_/mL.

#### RT-qPCR for ZIKV detection

Total RNA was extracted from viral supernatants using QIAamp Viral Mini Kit (QIAGEN, Germany) following the manufacturer’s protocols. The reverse transcription quantitative polymerase chain reaction (RT-qPCR) was conducted using the QuantiNova Probe RT-PCR Kit (QIAGEN, Valencia, CA, USA) with amplification in the QuantStudio 5 thermal cycler (Thermo Fisher, MA, USA) as per the manufacturer’s protocol. The reaction mixture (total volume, 10 μL) contained 5.0 μL of QuantiNova Probe RT-PCR Master Mix 2x, 0.8 μM each primers Zika 1086 (5′-CCGCTGCCCAACACAAG-3′), Zika1162c (5′CCACTAACGTTCTTTTGCAGACAT-3′), 0.4 μM FAM-labelled 1107(5′AGCCTACCTTGACAAGCAGTCAGACACTCAA-3′) probe for ZIKV^41^, 0.1 μL of QuantiNova RT Mix, 0.05 μL of QuantiNova ROX Reference Dye and 3.5 μL of the RNA samples or RNA-free water for negative control. The reaction program consisted of a single cycle of reverse transcription for 15 min at 45 °C, followed by 5 min at 95 °C for reverse transcriptase inactivation and DNA polymerase activation, and then 45 cycles of 5 s at 95 °C and 45 s at 60 °C. The RNA copy numbers in each sample were estimated by comparing the cycle quantification (Cq) values to the standard curve made by serial tenfold serial dilutions of ZIKV transcript previously constructed. To prepare in vitro transcribed RNA from the gene region amplified by the RT-qPCR primer set used as a copy number control, the ZIKV consensus primers with the T7 promoter sequence (TAATACGACTCACTATAGGGAGA) added to the 5′ end of forward primer were used to amplify a segment of cDNA. The same size segment of RNA was transcribed from the cDNA using the MEGAscript T7 Transcription Kit (Ambion, Thermo Fisher Scientific), according to manufacturers’ instructions. Following in vitro RNA synthesis, absorbance of the solution at 260 nm was determined using a Nanodrop 2000 (Thermo Scientific). Finally, the copy of RNA (molecules/μl) was calculated as described by Faye et al.^42^.

#### Antiviral assays

##### Pre and post-treatment

Vero cells were seeded in 48-well plates one day prior to infection at a density of 2.5 × 10^4^ cells/well. The cells were incubated with the maximum non-toxic concentration of ouabain (20 nM and its decreasing dilutions (10, 5 and 2.5 nM) before (pre-treatment) and after (post-treatment) 1 h of ZIKV PE243 strain at a multiplicity of infection (MOI) of 0.1. Then, cells were incubated at 37 °C in 5% CO_2_ for 120 h. Controls included mock, infected non-treated cells and as positive control, a drug previously identified by our group as a potent ZIKV inhibitor was used, the thiopurine nucleoside analogue 6-methylmercaptopurine riboside (6MMPr, 60.5 µM)^21^. At 120 h post infection (hpi), the cytopathic effect (CPE) was evaluated in both assays (pre and post-treatment) using an inverted microscope (AE2000 binocular microscope, Motic, Hong Kong) and pictures were taken using a smartphone. The cell supernatant was harvested and was stored at − 80 °C until analysis by TCID_50_ and RT-qPCR.

##### Virus inactivation assay

To analyze the inactivation activity of ouabain, a viral suspension containing ZIKV PE243 strain at a MOI of 0.1. was incubated with equal volume of the different concentrations of ouabain for 1 h at 37 °C, as previously described by Moghaddam et al.^43^. Then, viral titration was performed by the TCID_50_ method.

##### Anti-adsorption activity

Briefly, Vero cells were cultivated in 48 well plates at the density of 2.5 × 10^4^ cells/well one day prior to the assay. Cells were infected with ZIKV (MOI: 0.1) in the presence or absence of different concentrations of ouabain and incubated at 4 °C (permitting virus binding but not entry) for 1 h for virus adsorption, as previously described^43^. Then, cells were washed with sterile phosphate-buffered saline (PBS) twice and overlaid with DMEM. After 120 h, the cell supernatant was harvested and titrated by the TCID_50_ method.

##### Virus internalization inhibition

Vero cells were cultivated in 48 well plates at the density of 2.5 × 10^4^ cells/well one day prior to the assay. The microplate was incubated with ZIKV (MOI: 0.1) at 4 °C for 1 h to virus attachment. After 1 h adsorption, unabsorbed viruses were removed by washed with PBS and the cells were incubated at 37 °C (facilitating virus entry) for 1 h in the presence or absence of different concentrations of ouabain. Then, cells were washed with PBS and treated with 0.1 ml of citrate buffer (Citric acid 40 mM, KCl 10 mM, NaCl 135 mM, pH 3) for 1 min to inactivate adsorbed viruses but not internalized, as previously described^43^. The cells were overlaid with DMEM and incubated in standard conditions for 120 h. After that, the cell supernatant was harvested and titrated by the TCID_50_ method.

#### Statistical analysis

Data are expressed as mean ± SD and evaluated by one-way analysis of variance (ANOVA) followed by Tukey’s test, using the software GraphPad Prism v.6.0 for Windows (GraphPad Software, La Jolla, CA). Values of IC_50_ (half-maximal inhibitory concentration), CC_50_ (50% cytotoxicity concentration) and CC_20_ (20% cytotoxicity concentration) were calculated by linear and nonlinear regression using GraphPad Prism software. The selectivity index (SI) was obtained by calculating the ratio of the CC_50_ and IC_50_ values. Results were considered statistically significant when *P* < 0.05.

### Theoretical methods

The relative binding affinity between ouabain and non-structural proteins (NSP) of ZIKV was predicted using molecular docking and molecular dynamics simulations in three main steps: (i) the X-ray structures of three molecular targets of ZIKV that were co-crystallized with small-molecules were downloaded from the Protein Data Bank (PDB); (ii) the center of mass of the small-molecules in each binding sites of the receptors was used to determine the location of the binding pockets in each target, and then, the binding pose of the ouabain was predicted using the genetic algorithm (GA) of the GOLD software^44^; at last (iii) a post-docking analysis was carried out by means of molecular dynamics in order to refine the binding poses, as well as to re-scoring the relative binding affinity.

#### Preparation of the receptors

According to the work of Nandi et al.^5^, the following NSPs of ZIKV are considered the main targets for drug development: (i) NS3 Helicase, (ii) NS5 Methyltransferase (Mtase) and (iii) NS5 RNA-dependent RNA polymerase (RdRp). There are many X-ray structures of these receptors in the PDB, however, to the aim of this study we downloaded only geometries that were co-crystallized with small-molecules: NS3-Helicase (5RHX, 5K8T, 5RHV, 5RHP, 5RHM, 5RHR, 5RHN, 5RHG, 5RHK, 5RHQ, 5RHI, 5RHJ, 5RHL, 5RHU, 5RHW, 5RHO, 5RHS, 5RHT)^45^; NS5-Mtase (5WZ2^46^) and NS5-RdRp (5WZ3^46^). Using the PyMOL software^47^, the structures corresponding to each target were aligned considering the backbone atoms and the center of mass (COM) of the co-crystallized ligands were computed. Also, for the NS3-Helicase structures, we calculated the RMSD between the backbone of the structures, and verified that such structures present a low rate of conformational variation (RMSD < 1). In this case, we used the resolution of the structure as a criterion of choice. In summary, the receptors used for the molecular docking were: 5RHQ (NS3-Helicase), 5WZ2 (NS5-Mtase), and 5WZ3 (NS5-RdRp). Then, the selected structures for each molecular target were saved to an individual PDB file, and then, inspected for gaps in its backbone, whereas ligands, waters and ions were removed. The MODELLER software was used to insert missing residues. Further, the remaining structure was submitted locally to the PDB2PQR software^48^ in order to predicts the residues protonation at the pH 7.4 and hydrogens were properly added.

#### Preparation of the ligand

The 3D structure of ouabain was extracted from PDB 3A3Y^49^ to another file in PDB format. Then, using the OpenBabel package, the structure was converted to an individual MOL2 file and the partial charge of atoms determined by Gasteiger’s scheme.

#### Molecular docking

The genetic algorithm of the GOLD software^44^ (version 5.8.1) was used in standard mode to predict the ouabain binding pose in the binding pockets of the selected structures. The location of these pockets was determined considering the Euclidean distance between the centers of mass obtained in the “Preparation of the receptors”. In general, Euclidean distances ≃ 0 comprises to small-molecules that were co-crystallized in the same binding pocket. In this case, an average of the COM coordinates was considered instead of individual coordinates. These parameters were passed to the “Point” function of the GOLD configuration file, considering as active residues only those within cutoff radius of 12 Å.

##### Molecular dynamics simulations

The NSP-ouabain complexes obtained in the molecular docking step were subjected to molecular dynamics (MD) simulations. For this, the geometries of the NS3-Helicase, NS5-Mtase and NS5-RdRp were parameterized according to the FF14SB force field^50^ by using the tLeap module available in the AMBERTOOLS package. The ouabain structure, in turn, was parameterized according to the generalized AMBER force field (GAFF)^51^; the partial charge of the atoms was calculated by means of ANTECHAMBER module (also included in AMBERTOOLS package) using the AM1-BCC method. Further, the NSP-ouabain complexes were inserted in a cubic water box of 20 Å containing TIP3P waters and ions (0.15 M NaCl). MD simulation was performed by using the NAMD program^52^ (version 2.13) with the following configuration parameters: (i) periodic boundary conditions; (ii) restriction of vibration for covalent bonds involving hydrogen atoms, HOH angles and the OH bond distance of TIP3P water molecules (SHAKE algorithm); (iii) time step equal to 2 fs; (iv) electrostatic interaction cutoff of 12 Å for all steps of the simulations and (v) Particle Mesh Ewald (PME) method was used for long range electrostatic interaction. The starting geometry was submitted to minimization, heating (from 0 to 310 K) and pressurization steps. Then, the resulting geometry was submitted to the equilibrium step (NPT ensemble) during 10 ns; temperature and pressure were maintained constant along the simulation using the Langevin’s barostat and termostat with 1 atm and 310 K, respectively; frames were captured every 5 ps. The MD trajectories analysis were carried out using the CPPTRAJ^53^ software.

#### MM/PBSA

The MM/PBSA procedure included in the AMBERTOOLS package (MMPBSA.py)^54^ was applied to predict the relative binding free energy (∆*Gbind*). For this, 21 frames from the last 2 ns of the equilibrium MD simulation were used. In addition, the MM/PBSA parameters were adjusted according to the work of Wang et al.^55^: (i) modern non-polar solvent model; (ii) grid spacing equal to 0.50 Å and (iii) internal dielectric constant equal to 4; the remaining parameters were maintained in standard mode.

#### Binding enthalpy calculations

Single-point quantum chemistry calculations were performed in order to measure the binding enthalpy of the complexes, using PM7^56^, a semiempirical quantum chemical method. The MOPAC^57^ software was used according to the following parameters: (i) linear scaling algorithm MOZYME^58^; (ii) SCF convergence criteria in default configuration and cutoff radius of 9 Å; (iii) COSMO implicit solvent field with a relative permittivity of 78.4 Å; and (iv) an effective solvent molecule radius of 1.3 Å. Such calculations were performed for 21 frames from the last 2 ns of equilibrium MD simulation. Equation (1) was used to calculate the binding enthalpy.

1 $$\Delta {H}_{bind}=\Delta {H}_{f}^{complex}-\left(\Delta {H}_{f}^{receptor}+\Delta {H}_{f}^{ligand}\right).$$

## Data Availability

The data used to support the findings of this study are available from the corresponding author upon request.
